# A Comparison of Tests for Detecting Prior Exposure to *Coxiella burnetii* for Use with Q-VAX in Australian Human Q Fever Vaccination

**DOI:** 10.3390/vaccines13060615

**Published:** 2025-06-06

**Authors:** Stephen Graves, Jennifer Robson, Anja Scholzen, Richard Dzeng, Francisca Powell-Romero, Jennifer Evans, John Stenos, Meg Jeppesen, Milou L. C. E. Kouwijzer, Jordi Lankhof, Susan Raju Paul, Tatiana Proboste Ibertti, Lauren Ball, Helen Powell, Stephanie Wilkinson, Evi van Schuppen, Willemijn J. Anker-Op den Brouw, Rowland Cobbold, Anja Garritsen, Mark C. Poznansky, Ann E. Sluder

**Affiliations:** 1 Australian Rickettsial Reference Laboratory, Geelong, VIC 3220, Australia; graves.rickettsia@gmail.com (S.G.);; 2 Sullivan Nicolaides Pathology, Brisbane, QLD 4006, Australia; 3 Innatoss Laboratories B.V., 5342 AT Oss, The Netherlands; 4 Vaccine and Immunotherapy Center, Massachusetts General Hospital, Boston, MA 02114, USA; rdzeng@mgh.harvard.edu (R.D.);; 5 School of Veterinary Science, University of Queensland, Gatton, QLD 4343, Australiarowland.cobbold@scu.edu.au (R.C.)

**Keywords:** Q fever, *Coxiella burnetti*, vaccine eligibility, interferon-γ release assay (IGRA), immunity

## Abstract

Background/Objectives: Q-VAX vaccine, approved in Australia, prevents Q fever. However, individuals with prior *Coxiella burnetii* (*Cb*) infection have an increased risk of adverse reactions, requiring pre-vaccination screening by an intradermal hypersensitivity skin test for cell-mediated immune memory and a serological assay for anti-*Cb* antibodies. The week-long interval for skin test assessment limits efficient vaccination. This study evaluated a standardized interferon-γ release assay (IGRA) as a potential skin test alternative. Methods: Immune assays were compared in Australian populations with different incidences of prior *Cb* exposure. Cell-mediated immunity was assessed by the Q-VAX skin test and IGRA. Serological status was evaluated with established diagnostic assays. Hypothetical vaccine eligibility decisions using combined IGRA and serology results were compared with actual clinical decisions made using current guidelines. Results: All tests performed better in detecting prior infection than in detecting prior vaccination. Only the IGRA identified all individuals with a known history of Q fever. Agreement between the skin test and IGRA was limited. Moderate agreement was observed between hypothetical vaccine eligibility determinations based on IGRA plus serology results and actual clinical decisions. IGRA-positive but serology- and skin test-negative individuals received Q-VAX without clinically significant side effects, suggesting that elevated IGRA responses alone are not predictive of susceptibility to vaccine reactogenicity. Conclusions: The IGRA is not yet a suitable skin test replacement when assessing eligibility for Q fever vaccination, despite the significant limitations of the latter. We offer recommendations for designing future studies that might allow the development of appropriate guidelines for IGRA use in vaccine eligibility screening.

## 1. Introduction

Q fever, a zoonotic disease caused by the intracellular Gram-negative coccobacillus *Coxiella burnetii (Cb)*, is primarily transmitted to humans through contact with infected ruminants such as sheep, goats and cattle, particularly during procedures that can generate aerosolized bacteria able to persist in the environment, placing farmers, abattoir workers, veterinarians and other animal care professionals at risk [1]. Q fever outbreaks have occurred in a number of countries, most notably in Australia and the Netherlands. *Cb* exposure is also a concern for military service members due to high seroconversion rates among troops serving in endemic regions [2]. Acute human infection with *Cb* remains asymptomatic in many individuals. Even when symptomatic, acute Q fever is often self-limiting, rarely lethal, and treatable with antibiotics [1,3]. However, it can lead to serious long-term consequences in a subset of those infected, even when asymptomatic during the initial acute infection. Of particular concern, 1–5% of acute cases progress to a localized, persistent infection characterized by endocarditis, infected aneurysms, and endovascular or bone and joint infections. If left untreated, chronic Q fever has a fatality rate of up to 60% [4,5]. In addition, up to 20% of symptomatic infections result in a debilitating post Q fever fatigue syndrome that can persist for a year or more [6,7]. Due to this potential for long-term debilitating effects from *Cb* infection, coupled with environmental stability, low infectious dose, and aerosol transmission, *Cb* has been deemed a potential biothreat agent [8].

Q fever can be effectively prevented by vaccination with Q-VAX, a formalin-inactivated whole cell vaccine used in Australia since 1989 [9,10]. Q-VAX remains the only Q fever vaccine approved for use in humans, but it has not received regulatory approval outside Australia due to an increased risk for adverse reactions to the vaccine by individuals who have had prior *Cb* exposure. The resulting side effects variably include fever, malaise, and injection site granulomatous reactions [11,12,13,14,15,16]. These reactions are believed to result from a delayed hypersensitivity reaction to *Cb* lipopolysaccharide [12,17,18,19]. Rates of reactions appear to be higher in women than in men [14,15].

Mild cases of Q fever may go undiagnosed, and pre-vaccination screening by both an intradermal hypersensitivity skin test for cell-mediated immune memory and a serological assay for circulating antibodies against *Cb* is recommended [9]. Only individuals negative for both tests are eligible for Q-VAX vaccination However, demonstrated concordance between serological and skin tests is limited [14,20], and a subset of vaccinated individuals experience adverse reactions despite being negative in the pre-screens [14,15]. In practice, several serological tests are used interchangeably, but the sensitivity of these varies among tests and between laboratories [21,22,23]. Further confounding vaccine pre-screening, antibodies decline over time [24,25,26,27]. In addition, the week-long interval required for the assessment of skin test responses is a disadvantage when timely vaccination is needed (e.g., during Q fever outbreaks or employment requirement for high-risk exposure occupations) or when distance is a barrier to a prompt return to clinic (e.g., in rural and Q fever endemic regions of Australia). A more streamlined test for vaccine eligibility would be advantageous in these settings.

The largest natural outbreak of Q fever recorded to date occurred in the Netherlands in 2007–2010 with an estimated 40,000 infections [28,29]. Studies of Dutch Q fever patients and vaccinees suggest that ex vivo measurement of interferon gamma (IFNγ) release by circulating immune cells in response to stimulation by whole cell antigen can provide a sensitive assessment of cell-mediated immune memory responses to *Cb* [30,31]. In a Dutch cohort of mostly elderly individuals with cardiovascular risk factors who received Q-VAX under an emergency authorization during the Q fever outbreak, those with high pre-vaccination *Cb*-specific IFNγ responses exhibited an increased likelihood of local reactions to the skin test and the vaccine [14]. In a population that experienced a high incidence of *Cb* exposure and infection during the Dutch Q fever outbreak [32], a standardized IFNγ release assay (IGRA), Q-Detect, was a more sensitive means of detecting past *Cb* exposure than was a standard serological immunofluorescence assay currently used for clinical diagnosis [33].

The Q-Detect IGRA is performed using whole blood and provides results within 24 h, in contrast to the week required to determine the outcome of the Q-VAX skin test, offering the possibility of streamlining the assessment of pre-existing cellular immune memory against *Cb*. In the present study, we compared methods of detecting prior exposure to *Cb* in populations with different incidences of prior exposure, including in groups being pre-screened for Q-VAX vaccination. Based on the results of this initial direct evaluation of the IGRA in the context of vaccine eligibility screening, we offer recommendations for the design of larger studies that might allow the development of appropriate guidelines for use of the IGRA in settings where logistic considerations may preclude efficient use of the skin test.

## 2. Materials and Methods

### 2.1. Ethics Statement

The human studies in 2021–2023 involving Australian veterinary students undergoing routine Q-VAX vaccination and in Australian populations with known prior exposure to *Cb* were reviewed and approved by the University of Queensland Human Research Ethics Committee (St. Lucia, Queensland, Australia, protocol 2020001442). All participants provided written informed consent. Although administering a skin test to individuals with known prior exposure is outside standard clinical practice, this was deemed acceptable by the ethics review, as the risk of harm was viewed as minimal and the skin test result could provide some benefit to participants by letting them know whether or not they still had an active cellular immune response against *Cb* and likely ongoing immunity to Q fever.

Attendees at the 9th Tick and Tick Borne Pathogen/1st Asia Pacific Rickettsia Conference held in Cairns, Australia, in 2017 were offered the opportunity to receive Q-VAX vaccination if eligible at an onsite vaccine clinic administered by an Australian doctor (S.G.). Conference clinic attendees gave verbal informed consent for subsequent comparative analysis of deidentified exposure histories and test results by the diagnostic laboratory that supported the clinic (Sullivan Nicolaides Pathology [SNP]).

### 2.2. Human Study Cohorts

#### 2.2.1. Vaccine Clinic Attendees with Varied Exposure Histories

The on-site vaccine clinic at the 9th Tick and Tick Borne Pathogen/1st Asia Pacific Rickettsia Conference held in Cairns, Australia, in 2017 screened twenty-five individuals for prior exposure to *Cb* by clinical history and clinical diagnostic tests (Q-VAX intradermal skin test, Q-Detect IGRA, serology) (Table 1, Figure S1A). Seven individuals who were deemed eligible for Q-VAX vaccination based on skin test and serology assay results received vaccination. Vaccinated individuals were not followed up to determine whether any vaccine reactions occurred, as direct follow up is not part of standard clinical practice and because most attendees departed overseas immediately after the end of the conference. These conference attendees are hereafter collectively referred to as the Cairns cohort.

#### 2.2.2. Enrolled Study Cohorts with Unknown Exposure Histories

First-year veterinary students at the University of Queensland (UQ) undergoing required Q-VAX vaccination were enrolled February thru May in each of two years (2021 and 2022), allowing comparison of tests for prior *Cb* exposure in two cohorts. The 2021 cohort has been described previously [34]. A total of 211 students were enrolled in the study during the pre-vaccination screenings (Table 2). As part of the occupational health screening, students completed a questionnaire that assessed the likelihood of prior exposure to *Cb* (Survey 1; Supplementary Methods). Consistent with the overall student population in the UQ veterinary programs, the gender balance of study participants was skewed toward female participants. Following expanded recruiting efforts in 2022, males represented a larger proportion of the 2022 cohort. A majority of study participants in both cohorts were in the 18–22 age group (Figure S1B,C).

#### 2.2.3. Enrolled Study Cohort with Known Prior Q Fever Vaccination or Disease

Study participants with documented prior exposure to *Cb* antigens through Q-VAX vaccination or confirmed Q fever were recruited in the Q fever-endemic Darling Downs region of southeast Queensland, Australia. A community study clinic for enrollment of individuals known to have had either Q fever or Q-VAX vaccination was held at a Sullivan Nicolaides Pathology (SNP) collection room in Toowoomba by Australian doctors (S.G. & V.M.). Additional Q-VAX vaccinees were recruited during a regular onsite clinic for abattoir workers, conducted by an Australian doctor (V.M.). Participants completed a questionnaire regarding known exposures to *Cb* or Q-VAX (Survey 1; Supplementary Methods). The enrolled study population is summarized in Table 3. These *Cb*-exposed study participants are hereafter referred to as the Toowoomba cohort.

The demographic features of the Toowoomba cohort were similar to those of the Cairns cohort. Compared to the student cohorts they were significantly older (median age 53, range of 18–78; Figure S1D,E) and were predominantly male (65% vs. 18%). Background surveys indicated that, compared to the students, fewer of the Toowoomba participants were born outside Australia and more of them had potential *Cb* exposure risk via working in abattoirs, working with ruminants, spending time on farms, assisting with animal births, or hunting activities. This *Cb*-exposed cohort reported no or minimal levels of pain from their skin tests, and none reported seeking medical assistance following the skin test.

### 2.3. Tests of Immune Responses to Coxiella burnetii

#### 2.3.1. Q-VAX Intradermal Skin Test

Each participant was administered a skin test using a freshly prepared 1:30 dilution of the “Skin Test Reagent” (CSL Seqirus, Parkville, Victoria, Australia) that was equivalent to a 1/1000th dose of the Q-VAX vaccine. It was given by intradermal injection of 0.1 mL into the volar surface of the mid-forearm. Skin tests were clinically assessed by palpation after 7 days by an Australian doctor experienced in test interpretation (S.G. or V.M.). In addition, for the student cohorts local and systemic reactions within seven days after skin test or vaccination were self-reported via an online survey (Survey 2; Supplementary Methods) administered through the SurveyMonkey platform (SurveyMonkey, San Mateo, CA, USA). Vaccine eligibility decisions were based on the clinical assessment of the skin test, in keeping with established clinical guidelines, and did not consider self-reported reactions.

Intravenous blood was collected from participants at the time of study enrollment for clinical laboratory evaluation of anti-*Cb* immune responses. Blood samples were drawn prior to administration of the skin test to avoid any impact of the skin test.

#### 2.3.2. Assessment of Serological Status

*Cb* in culture can transition from a virulent phase I form to avirulent phase II, the two phases differing in lipopolysaccharide structure [35,36]. Phase-specific serology is used in the diagnosis of Q fever infection and exposure [23,37,38]. Serology assessments were made using enzyme immunoassays (EIA; SNP in-house-developed diagnostic assays for IgG and IgM against *Cb* phase II antigen, accredited by the Australian National Association of Testing Authorities [NATA]). The presence of anti-*Cb* antibodies was also evaluated at SNP using more sensitive indirect immunofluorescence assays (IFAs) for IgG, IgM, and IgA against both phase I and phase II antigens (Vircell commercial diagnostic reagents supplied by Abacus Diagnostics, Australia), and by complement fixation tests (CFT) (Virion/Serion Complement Fixation Test System, Wurzburg, Germany) using both phase I and phase II antigens.

#### 2.3.3. Whole Blood IFNγ Release Assay (Q-Detect 1.0 and Q-Detect 2.0)

The cellular immune status of participants was evaluated with the Q-Detect IGRA, in which IFNγ release is measured following stimulation of whole blood with heat-killed whole *Cb* antigen. The Cairns conference clinic utilized the original Q-Detect (1.0) IGRA adapted from the protocol used in a previous study [31], standardized and optimized for high throughput using a liquid antigen solution [33]. IGRA assays for the student and Toowoomba cohorts utilized a simplified assay format that employed ready-to-use antigen stimulation tubes precoated with heat-killed whole *Cb* antigen (Q-Detect 2.0), which had been demonstrated to perform with sensitivity and specificity equivalent to the original assay format [33]. Assays were performed as described [33,34]. Assay results for participants with negative control (background) IFNγ levels > 40 pg/mL were scored as inconclusive. Results were scored positive if negative and positive controls met the quality cutoffs defined by the supplier (see [33]), the *C. burnetii*-induced IFNγ production was ≥10 pg/mL, and the stimulation index (SI = *C. burnetii*-specific response/background response) was ≥10. Assay results with a SI ≥ 3 and <10 were considered borderline. Positives with an absolute IFNγ production below 30 pg/mL were scored “low positive”.

### 2.4. Determination of Vaccine Eligibility

Participants having no evidence of positive response in the skin or serological tests, and following review of exposure history [39], were deemed eligible for Q-VAX vaccination by the study clinicians. Vaccine eligibility of the Cairns clinic attendees is summarized above (Section 2.2.1). Three of the veterinary students had low-level positive phase II IgG EIA results and were therefore additionally assessed but tested negative by a phase II IgG IFA (titer < 1:25) and CFT (titer < 1:2.5). All 211 students were deemed eligible for Q-VAX vaccination based on a negative composite phase II IgG (EIA, IFA and CFT results), and the absence of skin test reactions. None of the Toowoomba study participants was eligible for vaccination based on their clinical history of either having had Q fever or Q-VAX vaccine.

### 2.5. Assessment of Post-Vaccination Immune Responses in Veterinary Student Cohorts

Blood samples were collected from Australian veterinary students after undergoing routine Q-VAX vaccination. Anti-*Cb* humoral immunity following Q-VAX vaccination was assessed at SNP with the same suite of serology assays used in the pre-vaccination screening (EIA, CFT, and IFA). Cellular immune responses after vaccination were evaluated with the IGRA. Of the 211 students enrolled in the study during the pre-vaccination screenings, 140 (66%) of the study participants returned for collection of post-vaccination blood samples (Table 4). In addition, local and systemic reactions within seven days after vaccination were self-reported via an online survey (Survey 3; Supplementary Methods) administered through the SurveyMonkey platform.

Most post-vaccination samples were collected within the target windows of 4–5 weeks (2021) or 5–7 weeks (2022) after vaccination (Figure S2). However, return of students for post-vaccination blood collection was disrupted by government-mandated COVID-19 restrictions (2021) and a major flood event in Brisbane (2022). Many of the impacted study participants were re-scheduled, though this resulted in some visits being outside the target windows.

### 2.6. Computational and Statistical Analyses

Data were analyzed with the scikit-learn library in Python 3.11. Figures were generated with matplotlib and seaborn. Serology and cell-based assay data were used to predict binary skin test results by logistic regression and random forest models with 3-fold cross validation. The best predictors were clustered by k-means clustering with k optimized by maximum silhouette score.

To examine relationships between survey data and outcomes, a Bayesian multinomial logistic regression model was fitted in R version 4.3.2 using the *brms* and *cdmstanr* packages [40,41], and specified using a categorical likelihood with a logit link function. Predictors for inclusion in the model were selected based on Pearson’s correlation coefficients, whereby a single predictor was selected among pairs with correlations greater than 0.6. Normally distributed priors with a mean of 0 and standard deviation of 1 were specified for the regression coefficients. Odds ratios were calculated by taking the exponential of model estimates and 95% highest posterior density confidence intervals.

Statistical analysis was performed using GraphPad Prism v10 (San Diego, CA, USA), unless otherwise indicated in a corresponding figure legend. Calculation of Cohen’s kappa was as described [42]. Kappa values were interpreted for the corresponding level of agreement as follows [43]: <0.2, poor; 0.21–0.40, fair; 0.41–0.60, moderate; 0.61–0.80, good; >0.80, very good.

## 3. Results

### 3.1. Pilot Comparison of Tests for Prior Exposure to Coxiella burnetii

Evaluation of markers of *Cb* exposure in vaccine clinic attendees at the 9th Tick and Tick Borne Pathogen/1st Asia Pacific Rickettsia Conference (held in Cairns, Australia, in 2017) compared the detection of prior exposure to *Cb* by the Q-Detect IGRA, by the intradermal skin test used for pre-vaccination screening, and by four clinically used serological assays for anti-*Cb* antibodies. Of 25 vaccine clinic attendees (the Cairns cohort), one had known prior exposure to *Cb* antigens due to prior Q fever infection and seven had already received Q-VAX vaccination in the past. Only the IGRA successfully identified all eight of these individuals (Table 5).

### 3.2. Pre-Vaccination Tests for Prior Exposure to Coxiella burnetii in Australian Veterinary Students

**Figure 1:**
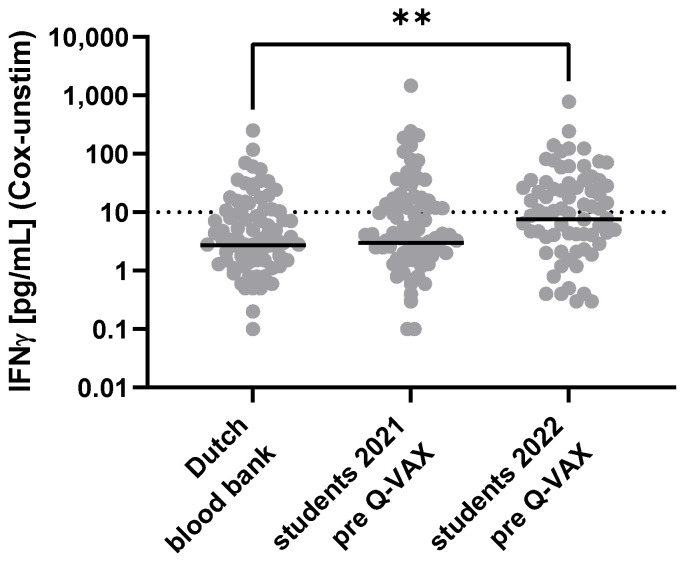
IGRA responses in populations with no recorded history of exposure to *Coxiella burnetii*. *Cb*-specific responses were assessed using Q-Detect 2.0 for 98 blood bank donors from a low incidence region of the Netherlands [33] and 96 (2021) and 115 (2022) Australian veterinary students during pre-screening to determine eligibility for Q-VAX vaccination. Dotted line indicates the technical IGRA positivity cutoff of 10 pg/mL [33]. Solid lines denote the median value in each group. Responses were compared between groups using the Kruskal–Wallis test followed by Dunn’s post hoc multiple comparison test for nonparametric data. Asterisks (**) indicate 0.001 < *p* ≤ 0.01; all other between-group differences were non-significant (*p* > 0.05).

The observations from the Cairns conference vaccine clinic suggested that the Q-Detect IGRA might offer a more sensitive means of detecting prior exposure to *Cb* antigens than current standard tests. To evaluate this possibility in a larger study, markers of prior *Cb* exposure were evaluated in students receiving required Q-VAX vaccination upon matriculation at the University of Queensland (UQ) Veterinary School in two consecutive years (2021, 2022). None of the study participants in either enrolled cohort experienced reactions to the Q-VAX skin test that were deemed positive by the study clinician or the UQ Health Services, and none had pre-vaccination serology test results that contraindicated vaccination. Vaccination was judged clinically appropriate for all study participants based on test results, age, and risk profiles. The range of IGRA responses observed for both study cohorts was similar to that observed in a cohort of Dutch blood bank donors from a region in the Netherlands with a low incidence of Q fever (Figure 1).

Participants self-reported varying degrees of short-term moderate reactions (redness, pain, swelling) in the surveys administered following the skin test (Survey 2) and vaccination (Survey 3) (see Supplementary Methods for survey questions). Symptoms were reported more frequently after vaccination than after the skin test (Figure S3). There were no definitive associations between any single self-reported response to the skin test or vaccination and the pre-vaccination IGRA responses (see example in Figure S4). Whether the short-term reactions to vaccination were limited to individuals with preexisting adaptative immune responses to *Cb* or primarily reflect innate responses to Q-VAX components remains unclear.

Based on the IGRA specificity of 82% estimated from the Cairns conference vaccine clinic observations (Table 5), we might anticipate 17 potential false positives in the 2021 student cohort and 21 in the 2022 cohort. Using the established Q-Detect IGRA positivity criteria, 72 (26/96, or 27%, in 2021; 46/115, or 40%, in 2022) of the study participants were scored as IGRA+. There was no correlation between the IGRA results and a composite score of self-reported risk factors for potential prior exposure (Figure S5). Therefore, the question of whether these pre-vaccination IGRA+ results represent false positives or are true indicators of prior subclinical *Cb* exposure remains unanswered.

### 3.3. Evaluation of Markers of Immune Responses to Coxiella burnetii in Individuals with Known Prior Exposure

Given the absence of positive skin test responses in the student cohorts, we pursued further comparison of the IGRA and the skin test in a smaller study enrolling individuals with known prior exposure to *Cb* antigen, via either infection or vaccination. Participants were enrolled both at an abattoir, where Q fever vaccination is a requirement for employment, and at a community clinic (collectively the Toowoomba cohort). In addition to the Q-VAX skin test, immune responses to *Cb* were assessed by the IGRA and by serology assays (Table 6). Three community clinic participants who had received Q-VAX vaccination > 10 years prior (>20 years prior for two of these), and who may have had little subsequent exposure to *Cb*, were negative in all tests. All other participants were positive for *Cb* immune responses in at least one test. Given their exposure histories and ongoing environmental and occupational exposure risks, this was to be expected.

**Figure 2:**
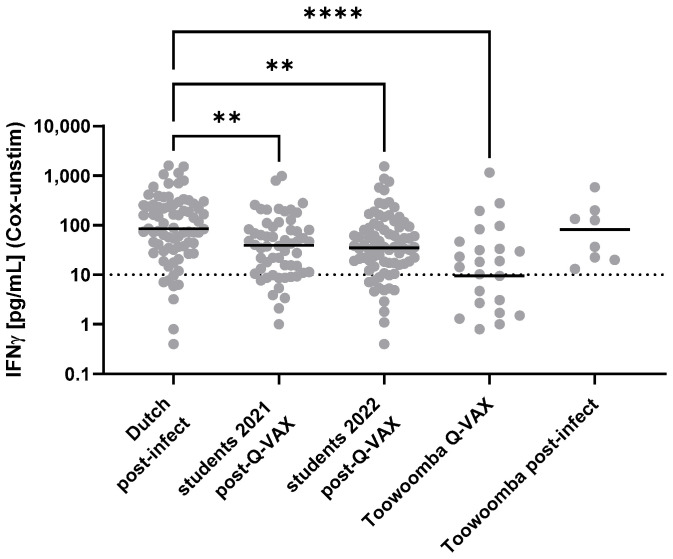
Cellular immune responses to *Coxiella burnetii* antigens in populations with known prior exposure. *Cb* specific responses were assessed using Q-Detect 2.0, with a technical positivity cutoff of 10 pg/mL [33]. Data are displayed on a log scale and hence zero values are not represented in the graphs. Individual IGRA responses and medians are shown for 75 Dutch Q fever convalescents, Australian veterinary students following Q-VAX vaccination (58 from 2021; 82 from 2022), and the Toowoomba study cohort (29 with prior Q-VAX vaccination; eight with prior Q fever). In the selection of the Dutch convalescent group in 2015, preference was given to individuals for whom the IGRA responses remained high to maximize the potential to detect *Cb* epitope-specific T cells [45]. Therefore, the level of cellular responses in this reference cohort is not representative of the full range of responses in individuals with prior *Cb* exposure. Responses were compared between groups using the Kruskal–Wallis test followed by Dunn’s post hoc multiple comparison test for nonparametric data. Asterisks indicate 0.001 < *p* ≤ 0.01 (**); *p* ≤ 0.0001 (****); all other between-group differences were non-significant (*p* > 0.05).

**Figure 3:**
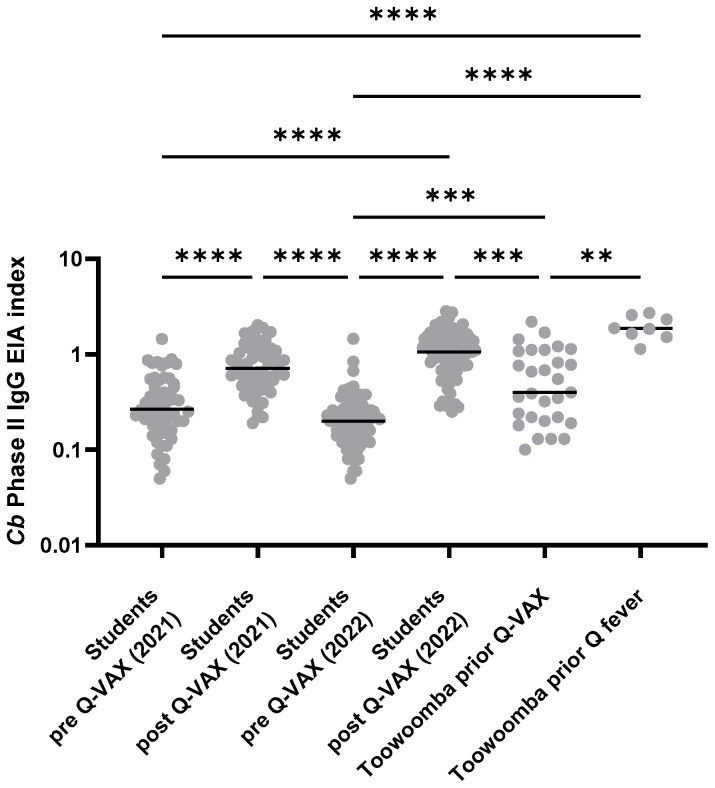
Circulating IgG antibodies to *Coxiella burnetii* phase II antigen. Serological status was determined by EIA. Results are shown for paired pre- and post-vaccination samples from 58 (2021) and 82 (2022) Australian veterinary students undergoing Q-VAX vaccination and for the Toowoomba study cohort (29 with prior Q-VAX vaccination; eight with prior Q fever). Data are shown for the phase II IgG EIA index (patient sample OD [Optical Density]/calibrator OD in serum). Lines show the median value. Levels were compared between groups using the Kruskal–Wallis test followed by Dunn’s post hoc multiple comparison test for nonparametric data. Asterisks indicate 0.001 < *p* ≤ 0.01 (**); 0.0001 < *p* ≤ 0.001; (***) *p* ≤ 0.0001 (****); all other between-group differences were non-significant (*p* > 0.05).

The range of IGRA responses in Toowoomba study participants was similar to that observed in other populations with known prior exposure to *Cb* antigen by infection or Q-VAX vaccination (Figure 2). The levels of circulating anti-*Cb* IgG varied more widely in Toowoomba study participants than in the students tested 4–6 weeks after vaccination (Figure 3), consistent with the former’s known wider variation in age and in time (often years) since infection or vaccination.

### 3.4. Comparison of the Intradermal Skin Test and Q-Detect IGRA as Indicators of Cellular Immune Memory of Prior Exposure to Coxiella burnetii Antigens

**Figure 4:**
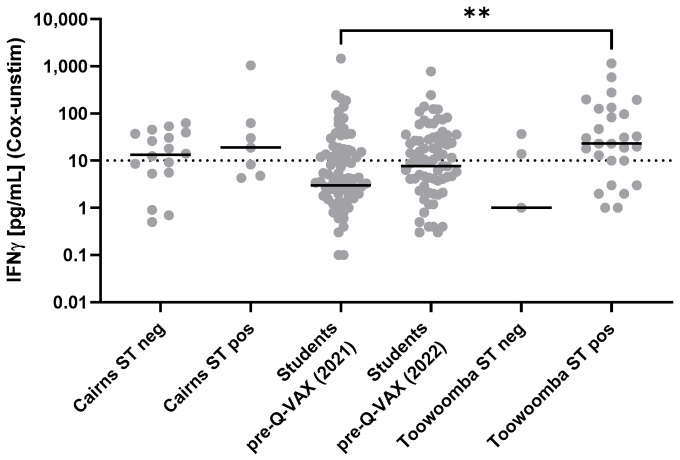
IGRA test responses in study participants with known Q-VAX skin test results. *Cb*-specific cellular immune responses were assessed using the Q-Detect IGRA with the technical positivity cutoff of 10 pg/mL (dotted line). Individual IGRA responses and group medians are displayed on a log scale and hence zero values are not represented in the graphs. Data for Australian veterinary students at vaccination pre-screening (96 from 2021; 115 from 2022) are those from Figure 1; all were skin test negative. The Cairns and Toowoomba cohort results are summarized in Table 5 and Table 6, respectively. ST neg—skin test negative; ST pos—skin test positive. Responses were compared between groups using the Kruskal–Wallis test followed by Dunn’s post hoc multiple comparison test for nonparametric data. Asterisks (**) indicate 0.001 < *p* ≤ 0.01; all other between-group differences were non-significant (*p* > 0.05).

The sensitivities of the IGRA and the Q-VAX skin test in detecting individuals with prior *Cb* exposure varied between the Cairns and Toowoomba study populations (Table 5 and Table 6). The reduced IGRA sensitivity in the Toowoomba study compared to that observed in the Cairns cohort was largely due to the failure to detect cellular immune memory > 10 years after Q-VAX vaccination in Toowoomba community participants who may have had limited subsequent exposure to *Cb* (Table 6). Although skin test-positive study groups had generally higher IGRA results, there was no clear separation between the IGRA response ranges of the skin test-negative and skin test-positive groups (Figure 4), and there was no direct correspondence of skin test and IGRA results for all individuals (Table 7). For a further assessment of the agreement between the skin test and IGRA as independent evaluators of cellular immune memory against *Cb*, we applied a Cohen’s kappa analysis as a measure of the observed agreement adjusted for that expected by chance [42,43]. Agreement between the two tests by this measure was poor (Table 7).

**Figure 5:**
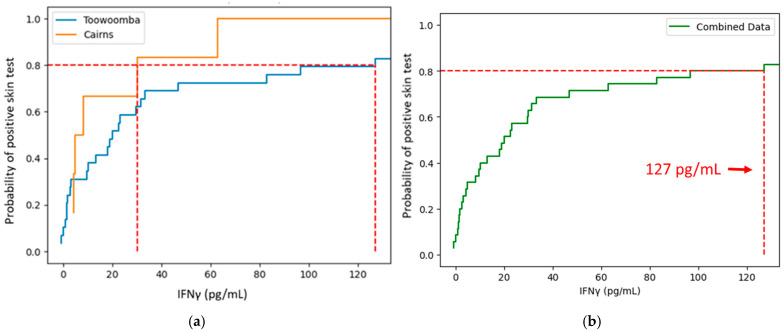
Empirical cumulative distribution function (ECDF) plots of IGRA results for skin test positive study participants. Expanded view of plots up to the 80% probability thresholds. Plots of the full data range are shown in Figure S6. (**a**) Results for Cairns and Toowoomba cohorts plotted separately. (**b**) Plot of the combined Cairns and Toowoomba results. Dashed lines indicate the thresholds for 80% probability of a positive skin test within the data plotted.

All the IGRA+ students were vaccinated, and none reported clinically significant reactogenic vaccine responses, suggesting that elevated IGRA responses alone are not predictive of susceptibility to vaccine reactogenicity. We therefore explored whether a higher positivity cutoff could be defined that might provide a more appropriate assay for identification of individuals likely to experience clinically significant reactogenicity to the Q-VAX skin test or vaccination. Empirical cumulative distribution functions (ECDFs) of positive skin test probabilities were calculated and plotted based on the IGRA assay results for skin test positive individuals in the Cairns and Toowoomba study cohorts (Figure 5 and Figure S6). These ECDFs were used to estimate that a tested 127 pg/mL IFNγ IGRA threshold corresponds with an 80% probability of a positive skin test in these cohorts (Figure 5b). Use of the 127 pg/mL IFNγ threshold reduced the number of skin test-negative individuals deemed IGRA+ but did not markedly improve the overall level of agreement between the two tests relative to that expected by chance, primarily due to the decreased number of skin test-positive individuals scored as IGRA+ (Table 8).

### 3.5. Comparison of the Intradermal Skin Test and Q-Detect IGRA for Determination of Q-VAX Vaccine Eligibility

We assessed how hypothetical vaccine eligibility decisions for our combined study cohorts using IGRA results in combination with serological testing might compare with the actual decisions made by the study clinician under the current clinical testing guidelines [39]. For eligibility determinations postulated using serology plus IGRA results, individuals with borderline test results (as defined by the supplier guidelines; see also [33]) were considered to be vaccine eligible if they were also serology negative. Individuals with positive IGRA results were deemed vaccine-ineligible regardless of serology status. Individuals with positive serology tests were deemed vaccine ineligible regardless of IGRA test results. The level of agreement between the actual clinical decisions and the hypothetical eligibility determinations based on combined IGRA and serology results (Table 9) was greater than that between the IGRA and skin test as independent indicators of cellular immunity (see Table 7). The level of agreement of IGRA-based and current eligibility improved using the higher ECDF-adjusted IGRA positivity threshold, primarily due to the increased number of individuals deemed vaccine eligible by both determinations (Table 10). Using the adjusted IGRA positivity threshold, >85% of study participants would have had the same vaccine eligibility using the IGRA in combination with a serology test as with the current clinical standard practice of skin test plus serology testing (overall % agreement in Table 10).

**Figure 6:**
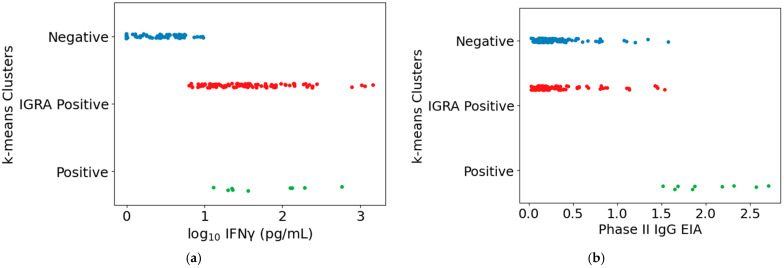
Study participant subpopulations as defined by k-means clustering (Table S3). Assay result distributions are plotted by subpopulation (cluster) for (**a**) IGRA and (**b**) EIA results for IgG against phase II *Cb*. “Positive” cluster: individuals positive in all four assays (IGRA and three serology assays—EIA, IFA, and CFT). “Negative” cluster: individuals negative or only weekly positive on all four assays. “IGRA Positive” cluster: individuals with measurable IGRA responses but no or only low levels of detectable antibodies against *Cb*.

To investigate further whether the combined results of the IGRA and the three serology assays (EIA, IFA, and CFT) distinguished specific subpopulations of study participants for which vaccination guidance might differ, unbiased k-means clustering was performed for all study participants (student, Toowoomba, and Cairns) based on the combined assay results. This analysis grouped participants into three clusters: individuals positive in all four assays (“positive” cluster); individuals negative or only weakly positive in all four assays (“negative” cluster), and individuals with measurable IGRA responses but no or only low levels of detectable antibodies against *Cb* (“IGRA-positive” cluster) (Figure 6 and Figure S7; Table S1).

**Figure 7:**
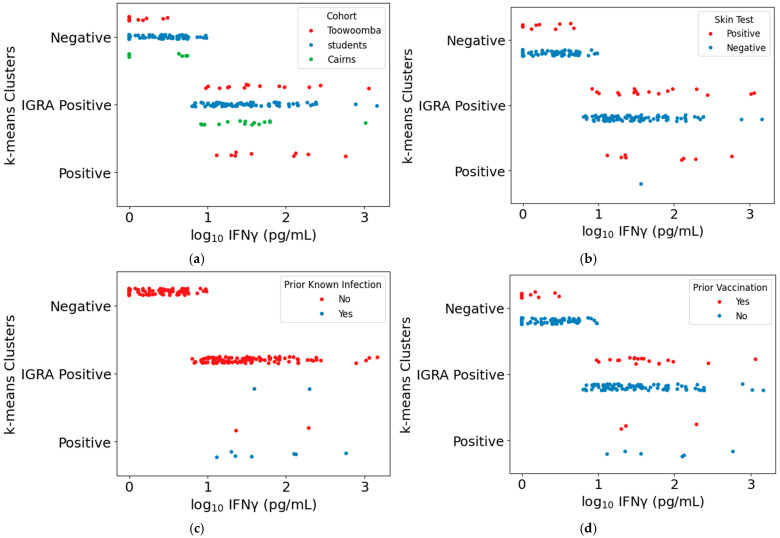
Demographic features of study participant subpopulations. For each graph, subpopulations as defined in Table S3 are shown on the Y axis and IGRA results are plotted on the X axis. Each subpopulation is subdivided as indicated: (**a**) Presence of study cohorts; (**b**) Individual Q-VAX skin test results; (**c**) Prior clinical diagnosis of Q fever; (**d**) Prior Q-VAX vaccination. “Positive” cluster: individuals positive in all four assays (IGRA and three serology assays—EIA, IFA, and CFT). “Negative” cluster: individuals negative or only weekly positive in all four assays. “IGRA Positive” cluster: individuals with measurable IGRA responses but no or only low levels of detectable antibodies against *Cb*.

The “positive” cluster comprised nine Toowoomba participants with high levels of IgG against phase II *Cb*, seven of whom had previously had Q fever; the other two were abattoir workers likely to have had occupational exposure to *Cb* in addition to vaccination (Figure 7a). The single skin test negative individual in the “positive” cluster (Figure 7b), a Toowoomba community participant reporting prior Q fever infection, was delayed in returning for skin test evaluation and thus may have had a false negative skin test result. None of these “positive” cluster individuals would be deemed eligible for vaccination.

The “negative” cluster included no individuals with prior known infection (Figure 7c). Eleven Toowoomba participants with prior vaccination were grouped in the negative cluster; at least five of these had received vaccination > 15 years ago (Figure 7d). A subset of these eleven were among the skin test-positive individuals in the “negative” cluster (Figure 7b), along with two Cairns vaccine clinic attendees having occupational risk of *Cb* exposure but no previous vaccination or known prior infection.

Seventeen of the individuals placed in the “IGRA-positive” cluster were skin test-positive individuals from the Toowoomba and Cairns cohorts (Figure 7b), of whom all but two of the Cairns conference vaccine clinic attendees had prior known infection or vaccination. Most skin test-negative individuals in the “IGRA-positive” cluster were students, all of whom received Q-VAX vaccination without adverse reactions. Of the twelve skin test-negative Toowoomba and Cairns cohort members in this cluster, five had previously received Q-VAX. The other seven were Cairns conference vaccine clinic attendees with no record of prior vaccination or infection, three of whom elected to receive Q-VAX.

## 4. Discussion

Here we have undertaken a comparative study of assay performances for detection of immune responses to *Cb* in Australian populations with varying incidences of prior exposure to *Cb*. A major goal of this study was to evaluate the potential for use of a standardized IGRA, Q-Detect, in Q-VAX vaccine eligibility screening. The sensitivity (the proportion of true positives that actually test positive) and specificity (the proportion of true negatives that actually test negative) of the IGRA were compared with those of established clinical serology assays and the standard Q-VAX skin test currently used in vaccine prescreening. In previous studies of a Dutch population that experienced a high incidence of *Cb* exposure and infection, ex vivo measurement of IFNγ release by peripheral lymphocytes provided a more sensitive means of detecting known *Cb* exposure up to 10–14 years after initial exposure than did serological assays or the standard Q-VAX skin test [30,31,33]. While the numbers of known Q fever convalescents in the current studies were small, the results are consistent with greater sensitivity of the IGRA compared to circulating antibody levels as markers of prior exposure to *Cb*.

Cellular immune responses to *Cb* and/or anti-phase II *Cb* IgG in peripheral blood can be detected in humans for at least 6–10 years after acute infection [24,33] or vaccination with inactivated whole cell *Cb* [46]. However, both immune indicators may wane below detectable levels in some individuals [26,46]. In the current study, all the tests performed better in detecting prior Q fever infection/exposure than in detecting prior vaccination, especially multiple years post-exposure. This is consistent with the longer-lasting immunological memory observed against other cellular pathogens following infection or administration of a live (attenuated) vaccine compared to that induced by inactivated or subunit vaccines [47].

The overall level of agreement between the skin test and the IGRA as independent indicators of cellular immune memory against *Cb* was limited. However, we note that the concordance between the two tests is comparable to that between IGRA and skin tests employed in the clinical diagnosis of tuberculosis, for which context- and region-specific guidelines for use are recommended (for a recent meta-analysis review, see [48]). Agreement among the different clinical serology assays as indicators of existing humoral immunity against *Cb* was also limited (Table 5 and Table 6). In the current study, a combination of CFT serology assessment and IGRA testing exhibited the best correlation with the existing standard skin test plus serology clinical vaccine pre-screening outcomes (Table 9 and Table 10). However, the standardized CFT reagents for clinical diagnostic testing are becoming increasingly difficult to obtain, limiting widespread routine use of this assay.

Definitive calculation of IGRA specificity was not formally possible as the study populations could not be assumed to be completely free of unrecognized prior *Cb* exposure. This is particularly relevant for interpretation of the IGRA results in the pre-vaccination screening of the UQ veterinary students. Based on a previous study of Q fever vaccination in UQ veterinary students [15], we had estimated that approximately 5% of students would present evidence of prior *Cb* exposure and not be eligible for vaccination, which would equate to 10–11 students in our combined pre-screened study cohorts of 211 students. The absence of confirmed clinical signals of prior exposure in the students enrolled in this current study could reflect the fact that only a minority reported known risk factors for previous *Cb* exposure (e.g., living on a farm that has sheep, cattle, or goats or working regularly with sheep, cattle, or goats).

Even though no individuals enrolled in the current UQ vaccination study had prior *Cb* exposure confirmed by either clinical record or currently used clinical assays (skin test and serology), 34% were IGRA+ using the technical IGRA positivity cutoff of 10 pg/mL. Whether these results are true indicators of prior subclinical exposure to *Cb* or false positives is unknown, limiting the ability to evaluate the specificity of the IGRA for use in pre-vaccination screening. More definitive assessment of IGRA specificity might be achieved by testing in New Zealand, where Q fever is presumed to be absent [49].

Moderate agreement was observed between vaccine eligibility decisions guided by current standard clinical practice and hypothetical eligibility determinations made based on IGRA results in combination with serology tests. However, these determinations were not concordant for all study participants. Ten serology-negative, skin-test-negative veterinary students would have been considered vaccine ineligible even with the higher IGRA+ threshold, leaving them at potential risk for Q fever. A similar or greater number of serology-negative, skin-test positive individuals from the Toowoomba and Cairns study cohorts (9–23 participants, depending on the serology test used) were IGRA-negative using the higher threshold, and would have been deemed vaccine eligible. Four of these serology-negative, skin-test positive participants had no record of prior vaccination or Q fever, though all had occupational risk factors for *Cb* exposure. One of these serology-negative participants who had a weakly positive skin test elected to receive a low dose of Q-VAX based on ongoing occupational risk. However, most of these participants had previously received Q-VAX, so that vaccination would potentially expose them to the risk of adverse vaccine reactions but could also boost their immunity to Q fever [30]. Consistent with this observation, the main finding from a logistic regression analysis of features of the clinical and exposure histories of study participants was that individuals with prior Q-VAX vaccination would have higher odds of being deemed vaccine eligible using the IGRA- and serology-based determination than under the current clinical testing paradigm (Table S2). In actual practice, vaccination would not be considered necessary for previously vaccinated individuals.

Initial evaluation of the data from the current study using a higher positivity cutoff for the IGRA suggested that this might provide a more appropriate threshold for determination of vaccine eligibility when used in combination with serology testing, especially in populations at risk of exposure to *Cb*. Additional assessment of the Q-Detect IGRA in larger vaccine studies, using a higher positivity threshold of 100–150 pg/mL, would be needed to determine if appropriate guidelines can be established for its use in vaccine eligibility screening. Alternatively, given the long-term health risks associated with Q fever, the potential for serology testing alone to provide an adequate assessment of existing immunity that would preclude the need for vaccination may also merit renewed consideration, particularly for those who will have ongoing occupational risk of exposure.

Vaccination decisions must balance the long-term health risks associated with contracting Q fever when withholding vaccination with the risks of severe adverse reactions to the Q-VAX vaccine. Notably, IGRA-positive (using the supplier-defined 10 pg/mL technical positivity threshold) but serology- and skin test-negative students received Q-VAX vaccination without suffering clinically significant adverse side effects, suggesting that elevated *Cb*-specific cellular immune responses (as measured by the IGRA) alone are not predictive of susceptibility to vaccine reactogenicity. These observations are consistent with those from vaccination of serology- and skin test-negative but IGRA-positive individuals at risk for chronic Q fever who received Q-VAX during the Dutch Q fever outbreak [14]. Interestingly, five serology-positive individuals, including two with borderline positive skin tests, were inadvertently vaccinated during the Dutch vaccination campaign but did not experience prominent adverse reactions, though three did report localized injection site reactions [14]. Recent mechanistic studies have indicated that CD4+ T cells and anti-*Cb* antibodies both contribute to vaccination site reactions in murine models of delayed-type hypersensitivity responses to Q fever vaccines [50,51,52]. If the same is true for reactogenic vaccine responses in humans, high risk of clinically significant vaccine reactions may occur primarily when levels of both anti-*Cb* antibodies and cellular immune responses are elevated. Vaccination may thus be acceptable for individuals at occupational risk of *Cb* exposure when detectable levels of these immune markers are borderline, as any vaccine reactions may be less debilitating than Q fever. Indeed, this is anticipated in the current guidelines for Q-VAX administration, which allow for vaccination of at-risk individuals with “indeterminate” or “borderline” serology or skin test results [39].

Retrospective studies of notified Q fever cases in Victoria and Queensland have reported Q fever in individuals who were positive in the standard clinical pre-screening paradigm and were thus deemed vaccine ineligible [53,54]. In the larger Queensland study, the incidence of Q fever in vaccine ineligible individuals was similar to that in a population that was untested and unvaccinated, suggesting that the unvaccinated individuals remained at risk of Q fever despite presumptive prior exposure to *Cb* [54]. These retrospective analyses did not distinguish among those who were considered vaccine ineligible tested positive by serology, the Q-VAX skin test, or both. These observations nevertheless highlight the continued need for improved means of identifying existing immunity to *Cb* while also distinguishing susceptibility to potential adverse reactions to Q-VAX.

## 5. Conclusions

This comparative study evaluated different tests of pre-existing immune responses to *Cb*. A primary objective was to assess whether the Q-Detect IGRA could replace the Q-VAX skin test in determining eligibility for Q-VAX vaccination when used in combination with any of several established serology assays. Within the study populations, neither the IGRA nor the skin test results exhibited complete concordance with the known prior exposure status of individual participants. The study results demonstrate that elevated IGRA responses alone are not predictive of clinically significant vaccine reactions, but do not address whether IGRA-positive, serology-positive but skin test-negative individuals would experience more pronounced vaccine reactions. Vaccine eligibility decisions projected based on the two different tests would leave different subsets of individuals at potential risk for infection or adverse vaccine reactions. Further clinical assessment of the comparative performance of the IGRA and skin test in larger vaccine studies would be needed to determine if scenario-based guidelines can be established for more effective use of each of these tests.

## Figures and Tables

**Table 1:** Cairns conference vaccine clinic attendees screened for prior *Cb* exposure.

Number	25
Gender	10 F/15 M
Median Age (yr)	49
Age Range (yr)	25–72
Prior Q-VAX (n)	7
Prior Q fever (n)	1

**Table 2:** Enrolled veterinary students.

	2021	2022
Number	96	115
Gender	83 F/13 M	83 F/32 M
Median Age (yr)	19	21
Age Range (yr)	18–39	18–36

**Table 3:** Enrolled Toowoomba study participants.

	Enrolled		Returned for Skin Test Reading	
	Abattoir	Community	Abattoir	Community
Number	23	14	20 ^a^	14 ^b^
Gender	7 F/16 M	6 F/8 M	7 F/13 M	6 F/8 M
Median Age (yr)	42	62	42	62
Age Range (yr)	18–63	26–78	18–63	26–78
Prior Q-VAX (n)	22	7	19	7
Prior Q fever (n)	1	7	1	7

^a^ Three of the abattoir participants did not return for skin test assessment. ^b^ Three community participants were unable to return 7 days post-skin test and were assessed for responses 9–13 days post-skin test. A positive skin test will remain positive for at least 14 days (V.M., personal communication, 2023).

**Table 4:** Student study population assessed for post-vaccination immune responses.

	2021	2022
Number	58	82
Gender	51 F/7 M	60 F/22 M
Median Age (yr)	19	21
Age Range (yr)	18–32	18–29

**Table 5:** Results of screening tests for immune responses to *Coxiella burnetii* antigens in Cairns conference vaccine clinic attendees.

	# Positive Tests/Total				
Screening Test	Prior Q-VAX ^a^	Prior Q Fever ^a^	No Exposure ^a^	Sensitivity ^b^ (CI ^d^)	Specificity ^c^ (CI ^d^)
Q-VAX skin test	2/7	1/1	4/17	38% (8.5; 76)	76% (50; 93)
Q-Detect IGRA	7/7	1/1	3/17	100% (63; 100)	82% (57; 96)
Phase II IgM EIA	0/7	0/1	0/17	0% (0; 37)	100% (80; 100)
Phase II IgG EIA	1/7	1/1	0/17	25% (3.2; 65)	100% (80; 100)
Phase II CFT	1/7	1/1	1/17	25% (3.2; 65)	94% (71; 100)
Phase II IgG IFA	4/7	1/1	0/17	62% (24; 91)	100% (80; 100)

^a^ Self-reported exposure histories of vaccine clinic attendees. ^b^ Proportion of eight “true positives” who actually tested positive. ^c^ Proportion of 17 “true negatives” who actually tested negative. ^d^ The 95% confidence intervals (CI) were calculated using the Clopper–Pearson exact method [44].

**Table 6:** Results of tests for immune responses to *Coxiella burnetii* antigens in Toowoomba cohort.

	# Positive Tests/Total				
	Abattoir		Community		
Screening Test	Prior Q-VAX ^a^	Prior Q Fever ^a^	Prior Q-VAX ^a^	Prior Q Fever ^a^	Sensitivity ^b^ (CI ^c^)
Q-VAX skin test	18/19 ^d^	1/1	4/7	6/7	85% (69; 95)
Q-Detect IGRA	13/21 ^e^	1/1	1/7	7/7	61% (43; 77)
Phase II IgG EIA	6/22	1/1	1/7	7/7	41% (25; 58)
Phase II CFT	19/22	1/1	3/7	7/7	81% (65; 92)
Phase II IgG IFA	10/22	1/1	2/7	7/7	54% (37; 71)

^a^ Self-reported exposure histories of study participants. ^b^ Proportion of these known “true positives” that actually tested positive. ^c^ The 95% confidence intervals (CI) were calculated using the Clopper–Pearson exact method [44]. ^d^ Three participants did not return for the skin test reading. ^e^ IGRA assay result for one participant was inconclusive.

**Table 7:** Agreement between skin test and IGRA results. The supplier-defined 10 pg/mL technical positivity threshold and scoring guidelines were used to determine IGRA results; borderline results by supplier guidelines were counted as negative for the purposes of this analysis. Inconclusive IGRA results (generally samples with a high background) for nine students were excluded from the analysis.

Cohorts	Both Positive n (%)	ST+ ^a^ IGRA− n (%)	ST− IGRA+ n (%)	Both Negative n (%)	Overall % Agreement	Cohen’s Kappa (95% CI)	Kappa Agreement Level
Student cohorts	0 (0)	0 (0)	67 (33)	135 (67)	67%	0.0 (−0.20; 0.20)	Poor
Cairns	2 (8)	5 (20)	2 (8)	16 (64)	72%	0.20 (−0.30; 0.70)	Poor
Toowoomba	20 (59)	9 (26)	2 (8)	3 (9)	68%	0.18 (−0.21; 0.58)	Poor
Combined cohorts	22 (8)	14 (5)	71 (27)	154 (59)	67%	0.18 (0.03; 0.32)	Poor

^a^ ST—skin test.

**Table 8:** Agreement between skin test and IGRA results using elevated IGRA positivity threshold. A 127 pg/mL threshold was used as a postulated clinical IGRA positivity cutoff (Figure 5b). Inconclusive IGRA results (generally samples with a high background) for nine students were excluded from the analysis.

Cohorts	Both Positive n (%)	ST+ ^a^ IGRA− n (%)	ST− IGRA+ n (%)	Both Negative n (%)	Overall % Agreement	Cohen’s Kappa (95% CI)	Kappa Agreement Level
Student cohorts	0 (0)	0 (0)	12 (6)	190 (94)	94%	0.0 (−0.55; 0.55)	Poor
Cairns	1 (4)	6 (24)	0 (0)	18 (72)	76%	0.19 (−0.37; 0.75)	Poor
Toowoomba	7 (20)	22 (65)	0 (0)	5 (15)	35%	0.08 (−0.14; 0.31)	Poor
Combined cohorts	8 (3)	28 (11)	12 (4)	213 (82)	85%	0.21 (−0.04; 0.40)	Fair

^a^ ST—skin test.

**Table 9:** Agreement between vaccine eligibility decisions made using IGRA results (using the supplier-defined technical positivity threshold) in combination with serology tests and actual clinical determinations. Participants from all study cohorts were included in the analysis.

	Vaccine Eligibility Determinations ^a^						
IGRA + Serology Combinations	Both No n (%)	Clin Yes IGRA No n (%)	Clin No IGRA Yes n (%)	Both Yes n (%)	Overall % Agreement	Cohen’s Kappa (95% CI)	Kappa Agreement Level
IGRA + EIA	28 (12)	62 (27)	13 (6)	129 (56)	68%	0.24 (0.10; 0.38)	Fair
IGRA + CFT ^b^	34 (15)	62 (27)	5 (2)	129 (56)	71%	0.34 (0.21; 0.48)	Fair
IGRA + IFA	32 (14)	62 (27)	10 (4)	128 (55)	69%	0.29 (0.16; 0.43)	Fair

^a^ Clinical determinations of vaccine eligibility (Clin) were made by S.G. ^b^ CFT (complement fixation test) results were unavailable for two participants.

**Table 10:** Assessment of vaccine eligibility decisions made using elevated IGRA threshold. Participants from all study cohorts were included in the analysis.

	Vaccine Eligibility Determinations ^a^						
IGRA + Serology Combinations	Both No n (%)	Clin Yes IGRA No n (%)	Clin No IGRA Yes n (%)	Both Yes n (%)	Overall % Agreement	Cohen’s Kappa (95% CI)	Kappa Agreement Level
IGRA + EIA	18 (8)	10 (4)	23 (10)	181 (78)	86%	0.44 (0.26; 0.62)	Moderate
IGRA + CFT ^b^	30 (13)	10 (4)	9 (4)	181 (79)	92%	0.71 (0.58; 0.83)	Good
IGRA + IFA	25 (11)	10 (4)	17 (7)	180 (78)	89%	0.58 (0.43; 0.73)	Moderate

^a^ Clinical determinations of vaccine eligibility (Clin) were made by S.G. For eligibility determinations postulated using serology plus IGRA results, individuals with IGRA results < 127 pg/mL (see Figure 5b) were deemed IGRA-negative and counted as vaccine eligible if they were also serology negative. Individuals with IGRA results > 127 pg/mL IFNγ were deemed vaccine-ineligible regardless of serology status. Individuals with positive serology tests were deemed vaccine ineligible regardless of IGRA test results. ^b^ CFT (complement fixation test) results were unavailable for two participants.

## Data Availability

All relevant data generated for this study are included in the manuscript and the Supplementary Files. The deidentified raw data supporting the conclusions of this manuscript will be made available by the authors upon request to any qualified researcher. Further inquiries can be directed to the corresponding authors.

## Supplementary Materials

The following supporting information can be downloaded at: https://www.mdpi.com/article/10.3390/vaccines13060615/s1, Figure S1: Age distributions of human study cohorts; Figure S2: Timing of post-vaccination blood sample collection for veterinary student cohorts; Figure S3: Self-reported symptoms following Q-VAX antigen exposure; Figure S4: Distribution of baseline *Coxiella burnetii*-specific IFNγ responses in individuals with different levels of self-reported injection site erythema; Figure S5: IGRA responses vs. composite score of self-reported risk factors for previous exposure to *Coxiella burnetii*; Figure S6: Empirical cumulative distribution function plots of IGRA results for skin test positive study participants (full data range); Figure S7: Study participant subpopulations as defined by k-means clustering. Table S1: Study participant subpopulations determined by k-means clustering of IGRA and serology assay results; Table S2: Odds ratios (ORs) and 95% credible intervals (CIs) from Bayesian multinomial logistic regression model comparing risk factors associated with vaccine eligibility groups. Supplementary Methods: Participant Questionnaires; Q Fever Risk Scoring.

## Abbreviations

CFT—Complement Fixation Test; CI—Confidence Interval; ECDF—Empirical Cumulative Distribution Function; EIA—Enzyme Immunoassay; IFA—Immunofluorescence Assay; IFN—Interferon; IGRA—Interferon—gamma Release Assay; SI—Stimulation Index; SNP—Sullivan Nicolaides Pathology; ST—Skin Test; UQ—University of Queensland.
