# Supplementary material for: A Comparison of Tests for Detecting Prior Exposure to Coxiella burnetii for Use with Q-VAX in Australian Human Q Fever Vaccination
Source: Vaccines (Basel). 2025 Jun 6;13(6):615. doi: 10.3390/vaccines13060615 (PMC12197614; doi:10.3390/vaccines13060615)
Supplement: Supplementary file 1 [file vaccines-13-00615-s001.zip › vaccines-3624985-supplementary.pdf]

vaccines

Supplementary Material

A Comparison of Tests for Detecting Prior Exposure to *Coxiella burnetii* for Use with Q-VAX in Australian Human Q Fever Vaccination

Stephen Graves [1], Jennifer Robson [2], Anja Scholzen [3,†], Richard Dzeng [4], Francisca Powell-Romero [5], Jennifer Evans [2], John Stenos [1], Meg Jeppesen [2], Milou L. C. E. Kouwijzer [3], Jordi Lankhof [3], Susan Raju Paul [4], Tatiana Proboste Ibertti [5], Lauren Ball [2], Helen Powell [2], Stephanie Wilkinson [2], Evi van Schuppen [3,‡], Willemijn J. Anker-Op den Brouw [3], Rowland Cobbold [5,§], Anja Garritsen [3], Mark C. Poznansky [4,*] and Ann E. Sluder [4,*]

1. Australian Rickettsial Reference Laboratory, Geelong, VIC 3220, Australia; graves.rickettsia@gmail.com (S.G.)
2. Sullivan Nicolaides Pathology, Brisbane, QLD 4006, Australia
3. Innatoss Laboratories B.V., 5342 AT Oss, The Netherlands
4. Vaccine and Immunotherapy Center, Massachusetts General Hospital, Boston, MA 02114, USA; rdzeng@mgh.harvard.edu (R.D.)
5. School of Veterinary Science, University of Queensland, Gatton, QLD 4343, Australia; rowland.cobbold@scu.edu.au (R.C.)

* Correspondence: mpoznansky@mgh.harvard.edu (M.C.P.); asluder@mgh.harvard.edu (A.E.S.)
† Current address: Byondis B.V., 6545 CM Nijmegen, The Netherlands.
‡ Current address: MSD Animal Health, 5831 AN Boxmeer, The Netherlands.
§ Current address: Veterinary Medicine, Southern Cross University, South Lismore, NSW 2480, Australia.

Supplementary Figures

(a)

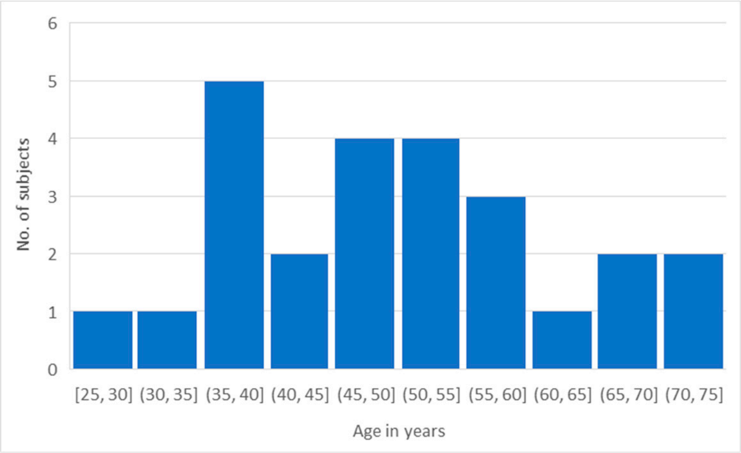

(b)

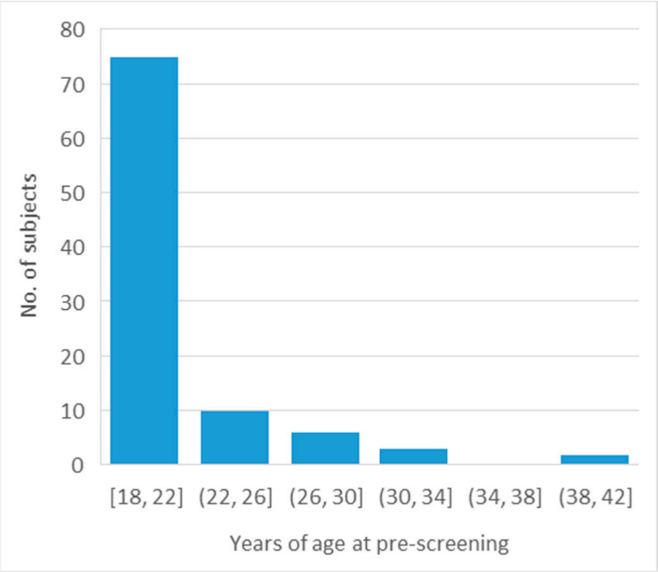

(c)

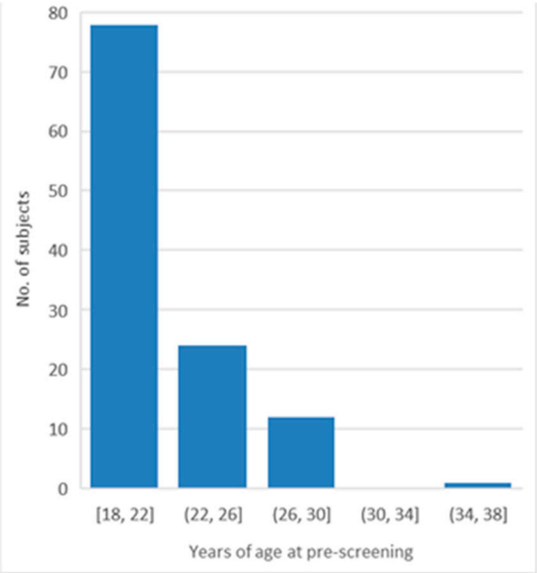

(d)

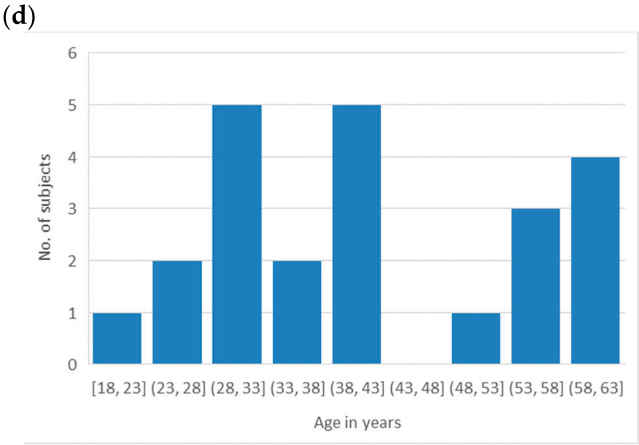

(e)

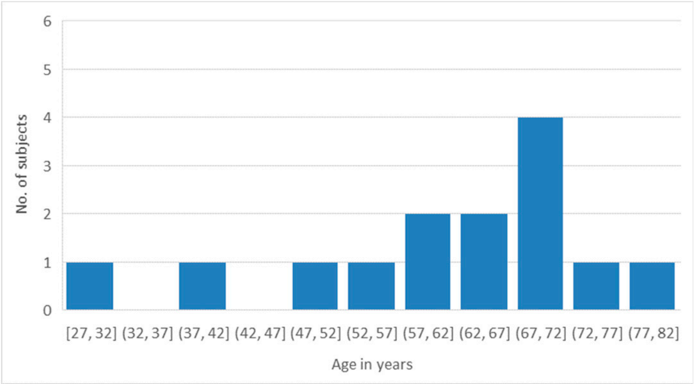

Figure S1. Age distributions of human study cohorts (**A**) 2017 Cairns conference vaccine clinic attendees; (**b**) Veterinary students enrolled in 2021; (**c**) Veterinary students enrolled in 2022; (**d**) Toowoomba study participants enrolled at abattoir vaccine clinic; (**e**) Toowoomba study participants enrolled at community testing clinic.

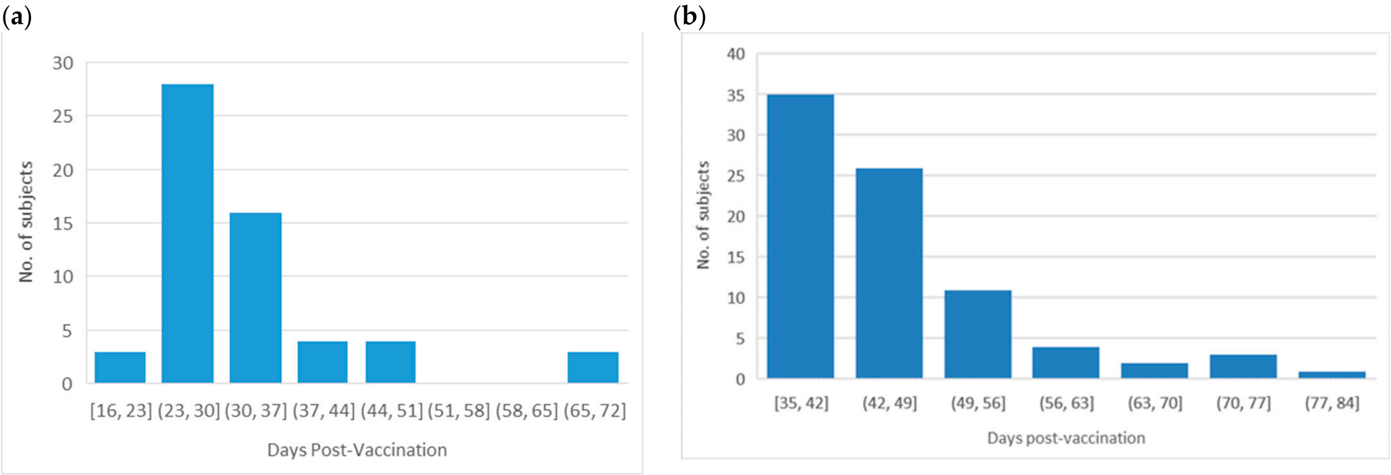

Figure S2. Timing of post-vaccination blood sample collection for veterinary student cohorts (**a**) Veterinary students enrolled in 2021 (median collection interval: 30 days post-vaccination); (**b**) Veterinary students enrolled in 2022 (median collection interval: 45 days post-vaccination).

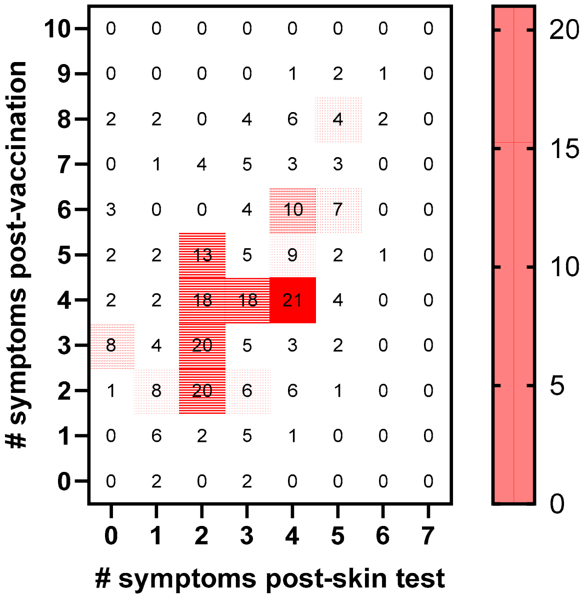

Figure S3. Self-reported symptoms following Q-VAX antigen exposure. Number of symptoms reported following administration of the Q-VAX Skin Test (Survey 2) or Q-VAX vaccine (Survey 3). Each cell in the grid indicates the number of participants reporting the corresponding number of symptoms; heat map scale is indicated in the bar to the right. Data from both student cohorts are included, but only for those individuals who responded to both surveys.

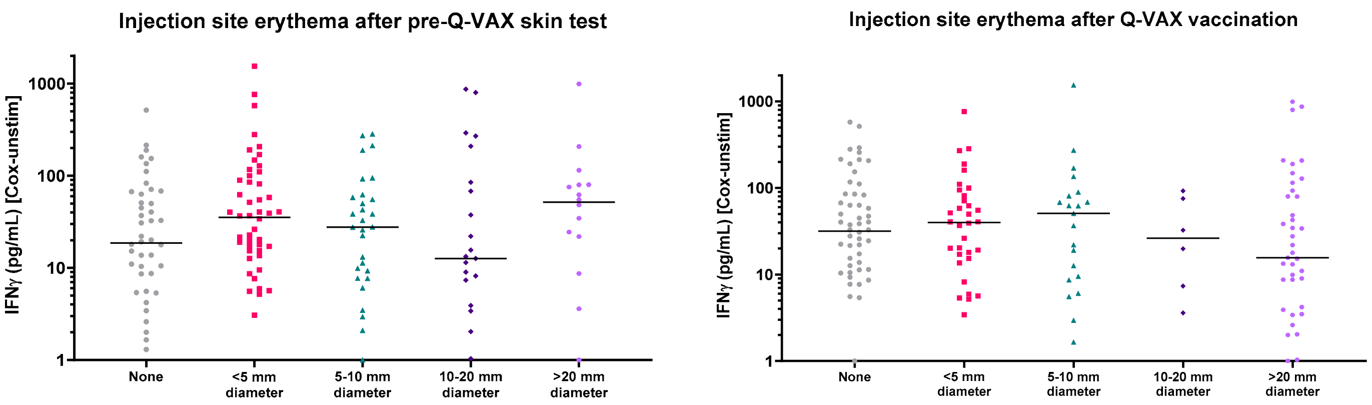

Figure S4. Distribution of baseline *Coxiella burnetii*-specific IFNγ responses in individuals with different levels of self-reported injection site erythema. Local and systemic reactions within seven days after skin test (left panel) or vaccination (right panel) were estimated and self-reported by students via an online survey administered through the SurveyMonkey platform (Momentive, Inc.); erythema at the injection site was the most commonly reported reaction (only the presence of induration and not erythema alone is interpreted as a clinically positive skin test reaction). Background corrected *Cb*-specific IFNγ responses are displayed on a log scale and hence zero and negative values are not represented in the graph. Individual results are represented using a scatter dot plot. Lines show the median value. Data from both student cohorts are combined.

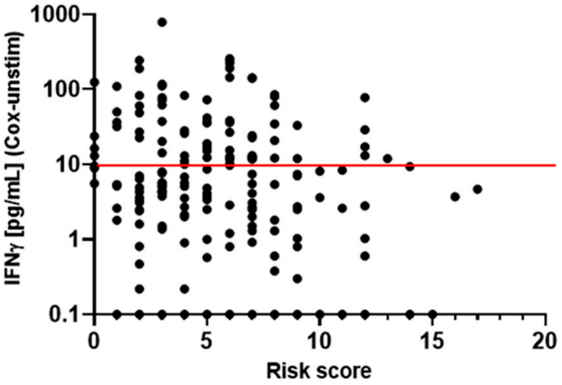

Figure S5. IGRA responses vs. composite score of self-reported risk factors for previous exposure to *Coxiella burnetii*. Data for both student cohorts are combined. The X axis plots composite risk scores derived from pre-screening Survey 1 (see **Supplementary Methods** for explanation of the risk scoring). IGRA test results are plotted on the Y axis; red line indicates the IGRA technical positivity cutoff of 10 pg/mL. There was no correlation between the risk score and IGRA results.

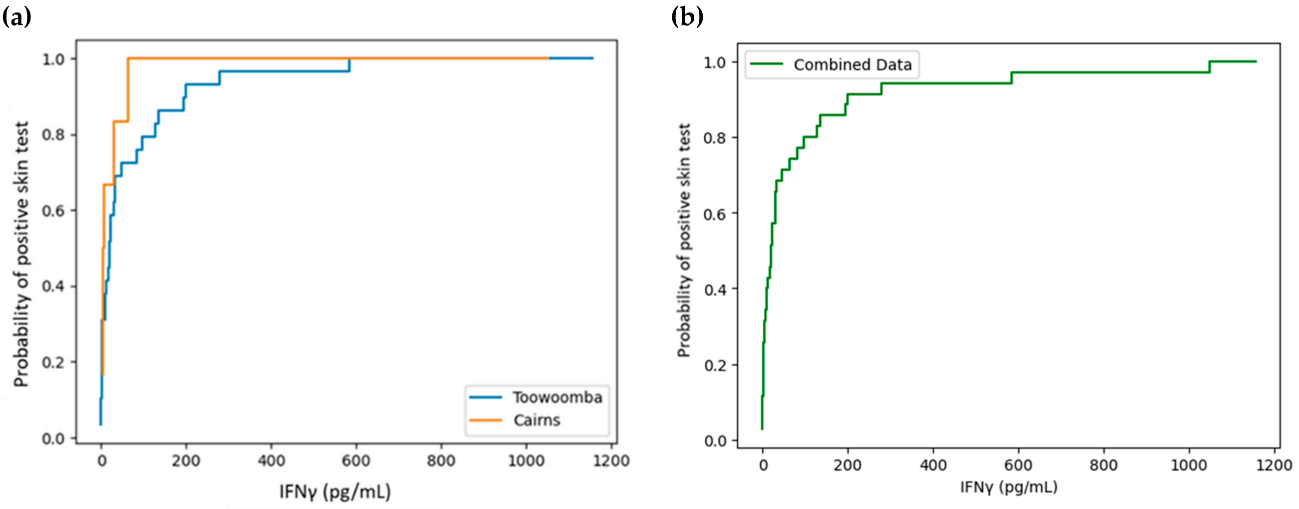

Figure S6. Empirical Cumulative Distribution Function (ECDF) plots of IGRA results for skin test positive study participants (full data range). **(a)** Results for Cairns and Toowoomba cohorts plotted separately. **(b)** Plot for combined Cairns and Toowoomba results.

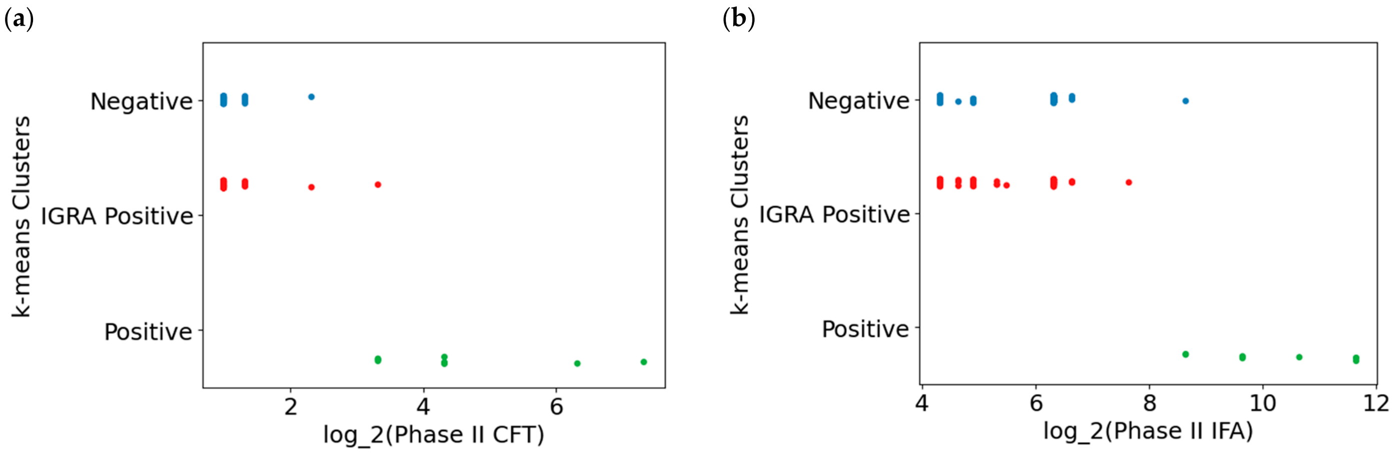

Figure S7. Study participant subpopulations as defined by k-means clustering (**Table S3**). Assay result distributions are plotted by subpopulation (cluster) for **(a)** CFT for antibodies against *Cb* and **(b)** IFA for IgG against phase II *Cb*. "Positive" cluster: individuals in all four assays (IGRA and three serology assays—EIA, IFA, and CFT). "Negative" cluster: individuals negative or only weekly positive in all four assays. "IGRA Positive" cluster: individuals with measurable IGRA responses but no or only low levels of detectable antibodies against *Cb*.

Supplementary Tables

Table S1. Study participant subpopulations determined by k-means clustering of IGRA and serology assay results. Subgroups of study participants were defined as k-means clusters based on individual IGRA and serology assays (IgG EIA, CFT, and IgG IFA) for antibodies recognizing phase II *Cb* antigen. Participants from all study cohorts (student, Toowoomba, Cairns) were included in the clustering. As linear data distributions improve k-means clustering, the IGRA data were $\log_{10}$ transformed and the CFT and IFA data were $\log_2$ transformed prior to clustering; EIA results were within linear range of the assay. Data were also [0,1] normalized to reduce the chance that variances in scale would distort the results. Optimal clustering was obtained with k=3 based on silhouette score assessment. Mean and standard deviation (SD) of assay results for individuals within each cluster are given.

Subpopulation (Cluster)	N	$\log_{10}$ IGRA (SD)	EIA (SD)	$\log_2$ CFT (SD)	$\log_2$ IFA (SD)
Negative in all assays	119	0.289 (0.304)	0.305 (0.267)	1.020 (0.142)	5.602 (0.986)
IGRA-positive, serology-negative	107	1.538 (0.509)	0.323 (0.328)	1.061 (0.269)	5.050 (0.927)
Positive in all assays	9	1.777 (0.562)	2.044 (0.425)	4.544 (1.394)	10.199 (1.236)

Table S2. Odds ratios (OR) and 95% credible intervals (CI) from Bayesian multinomial logistic regression model comparing risk factors associated with vaccine eligibility groups. Baseline group is eligibility based on all tests (n = 151).

Predictors	Clinically eligible (skin test-neg & serology-neg) N = 8		Eligible based on IGRA-neg & serology-neg N = 20		Ineligible N= 15	
	OR	95% CI	OR	95% CI	OR	95% CI
Prior vaccination	0.84	0.14; 5.09	**32.92**	**10.63; 105.69**	**8.11**	**2.44; 27.30**
Prior infection	0.90	0.12; 5.98	0.78	0.12; 4.44	**24.04**	**6.41; 89.31**
Risk score	0.93	0.72; 1.15	1.07	0.89; 1.27	**1.20**	**1.01; 1.44**
Born or lived in a country other than Australia	0.80	0.25; 2.58	0.45	0.14; 1.31	0.39	0.10; 1.36
Heard of Q fever	0.35	0.10; 1.11	1.54	0.49; 5.45	1.86	0.54; 7.18

Supplementary Methods

Participant Questionnaires

Survey 1

QVAX Immune Response Study – Participant Background

1. Your age in years ____________
2. Your gender
   ☐ Male
   ☐ Female
   ☐ Rather not say
3. Your current home postcode___________
4. Were you born in a country other than Australia or have you lived in another country for longer than 3 months?
   Yes ☐ No ☐
   If YES, please specify the country ____
   (list all if more than one)

5. Have you EVER worked…

At an abattoir	Yes ☐	No ☐
With sheep, cattle or goats or transported these animals	Yes ☐	No ☐
With animals other than sheep, cattle or goats. If yes, please indicate which animals, primarily _______________________________________	Yes ☐	No ☐

6. Have you EVER lived …

In a rural/country area (a locality with a population of fewer than 100,000 people, approximately) for longer than 3 months	Yes ☐	No ☐
On a farm that has sheep, cattle or goats	Yes ☐	No ☐
On a farm that grows crops commercially (e.g. vegetables, grain, fruit)	Yes ☐	No ☐
On a property that adjoined native bushland (e.g. national park)	Yes ☐	No ☐

7. Have you EVER …

Consumed unpasteurised milk or milk products (e.g cheese)	Yes ☐	No ☐	Unsure ☐
Assisted or been present at an animal birth or abortion<br>*If yes, specify type(s) of animals*______________________	Yes ☐	No ☐	Unsure ☐
Been involved in shooting/hunting/preparing hunted animals	Yes ☐	No	Unsure ☐

Worked with animals or animal products in any other way. Yes ☐ No ☐ ☐ Unsure ☐
If yes, please indicate how ___________________________

8. Please indicate your HIGHEST level of direct contact (e.g. touching) with any of the following AT ANY TIME DURING YOUR LIFE:

Frequency	Most days a week	A few days a week	Occasionally (at least 6 times a year)	Rarely (less than 6 times a year)	Never any contact
Sheep, cattle, goats	☐	☐	☐	☐	☐
Native animals	☐	☐	☐	☐	☐
Feral animals	☐	☐	☐	☐	☐
Pets	☐	☐	☐	☐	☐
Other animal 1 specify ___________________	☐	☐	☐	☐	☐
Other animal 2 specify ___________________	☐	☐	☐	☐	☐
Pond water	☐	☐	☐	☐	☐

9. Please indicate your smoking status (tick only one)
   Current smoker ☐
   Previous smoker ☐
   Never a smoker ☐
10. Are you taking immune suppressive medications or do you have other immune-modifying conditions or treatments? Yes ☐ No ☐
11. Have you had an MRI using a contrast agent within the last month? Yes ☐ No ☐
12. Have you ever received chemotherapy with a platinum-based drug? Yes ☐ No ☐
13. Before today, had you heard of a disease called Q fever? Yes ☐ No ☐
14. Have you ever been told by your doctor you had Q fever? Yes ☐ No ☐
   *If yes, What year did you have Q fever diagnosed (please estimate if unsure)?*________

15. Did you know there was a vaccine against Q fever (recommended for people at risk)?
   ☐ No, I did not know there was a Q fever vaccine
   ☐ Yes, and I have been vaccinated
   ☐ Yes, but I cannot recall if I have been vaccinated
   ☐ Yes, but I have NOT been vaccinated because I do not think I am at risk of Q fever
   ☐ Yes, but I have NOT been vaccinated because (*please provide details below*)

   ____

Survey 2

QVAX Evaluation Study - Post Skin Test Survey

1. **Did you experience pain at the injection site?**
   - ○ Yes
   - ○ No

   If yes, did the pain occur:
   - ○ On touch only
   - ○ When the limb was moved
   - ○ Continuously, with no need for touch or movement?

2. **Did you experience redness at the injection site?**
   - ○ Yes
   - ○ No

   If yes, please indicate the largest diameter of redness, as measured with a ruler.

   Diameter of redness in mm

3. **Did you experience swelling at the injection site?**
   - ○ Yes
   - ○ No

   If yes, please indicate the largest diameter of swelling, as measured with a ruler.

   Diameter of swelling in mm

4. **Did you experience itchiness at the injection site?**
   - ○ Yes
   - ○ No

   If yes, please indicate the severity of itchiness.

5. **How many days after the skin test did the injection reaction begin?**
   - ○ 1 day
   - ○ 2-5 days
   - ○ >5 days

6. **Did you seek medical attention for the injection site reaction?**
   - ○ No
   - ○ Yes, from the University Health Services
   - ○ Yes, from a General Practitioner

7. **Did you experience any other adverse events in the 4 weeks following the skin test?**
   - ○ Yes
   - ○ No

   If yes, please describe the symptoms below.

   [ ]

8. **If you have a late reaction to the skin test** (i.e. beyond 1 week, when this survey was administered), please contact Dr. Steven Graves (physician and Director of the Australian Rickettsial Reference Laboratory) on xx.

Survey 3

QVAX Evaluation Study - Post-Vaccination Survey

9. **Did you experience pain at the injection site?**
   - ○ Yes
   - ○ No

   If yes, did the pain occur:
   - ○ On touch only
   - ○ When the limb was moved
   - ○ Continuously, with no need for touch or movement?

10. **Did you experience redness at the injection site?**
    - ○ Yes
    - ○ No

    If yes, please indicate the largest diameter of redness, as measured with a ruler.

    Diameter of redness in mm

11. **Did you experience swelling at the injection site?**
    - ○ Yes
    - ○ No

    If yes, please indicate the largest diameter of swelling, as measured with a ruler.

    Diameter of swelling in mm

12. **How many days after the vaccine did the injection reaction begin?**
    - ○ 1 day
    - ○ 2-5 days
    - ○ >5 days

13. Did you seek medical attention for the injection site reaction?

- ○ No
- ○ Yes, from the University Health Services
- ○ Yes, from a General Practitioner

14. Did you experience fever in the 7 days following vaccination?

- ○ Yes
- ○ No

If yes, please indicate the highest temperature?

Temperature in °C

15. Did you experience headache in the 7 days following vaccination?

- ○ Yes
- ○ No

If yes, did you seek medical attention and/or self-medicate?

16. Did you experience lethargy or weakness in the 7 days following vaccination?

- ○ Yes
- ○ No

If yes, did you seek medical attention?

17. Did you experience joint pain in the 7 days following vaccination?

- ○ Yes
- ○ No

If yes, did you seek medical attention and/or self-medicate?

18. Did you experience any other adverse events in the 4 weeks following vaccination?

- ○ Yes
- ○ No

If yes, please describe the symptoms below.

19. If you have a late reaction to the vaccine (i.e. beyond 4 weeks, when this survey was administered), please contact Dr. Steven Graves (physician and Director of the Australian Rickettsial Reference Laboratory) on xx.

Q Fever Risk Scoring

Risk scoring was based on the answers given in Survey 1. Questions were divided into several parameters. For 11 parameters a score of either 0, 1 or 2 could be given, as summarized in Table below. Some survey parameters were not utilized in risk scoring, as noted in Table below.

**Parameter**	Risk Score			**Comments**
	**0**	**1**	**2**	
Age	17-25	25-39	40+	Increased age would increase risk, since older people have had more time to encounter Q-fever.
Gender	Female		Male	Males, due to their more frequent outdoor professions and more contact with animals, are more likely to contract Q-fever
Postcode				Parameter not included for two reasons. 1) Unable to clearly differentiate urban, regional and rural/remote postcodes. 2) Other survey questions were similar and provided clearer answers.
Origin country				Parameter not included. There is no reason to believe that living abroad would affect the likelihood to contact Q-fever. And even if this were true, it is impossible to indicate which countries would provide a higher risk.
Worked at an abattoir	No		Yes	Working at an abattoir would increase the risk.
Worked with sheep, cattle or goats or transported these animals				Parameter not included, since the parameter 'Level of direct contact with sheep, cattle and goats' is more specific.
Worked with animals other than sheep, cattle or goats				Parameter not included, since the parameter 'Level of direct contact with other animals' is more specific.
Living on a farm	No	In a rural/country area, on a farm that grows crops, on a property that adjoined native bushland	On a farm that has sheep, cattle or goats	Living on a farm that has sheep, cattle or goats would increase the risk and a score of 2 was given. However, living in a rural/country area could also increase the risk, since farm animals or other animals that could have Q-fever are relatively close by. The same applies to living on a farm that grows crops or living on a property that adjoined native bushland. Therefore, in this case a score of 1 was given.

Consumed raw milk	No	Unsure	Yes	Consuming unpasteurized increases risk. The risk would be dose dependent. However, since it is impossible to know how much unpasteurized milk was consumed, a score of 2 was given when answered with 'Yes'. When the answer was 'Unsure' a score of 1 was given.
Been present at an animal birth or abortion	No	Yes, other animals	Yes, ruminants	Being present at an animal birth or abortion increases risk. However, this is dependent on the type of animal. We manually evaluated the free form responses. All answers containing farm animals resulted in a score of 2. If the participant had been present at an animal birth or abortion of any other animal, the score was 1.
Been involved in shooting/hunting/preparing hunted animals	No	Worked with animal products in any other way	Yes	Being involved in hunting and/or preparing hunted animals increase risk. A score of 2 was given if the answer was 'Yes'. If the participant indicated having worked with animal products in any other way, a score of 1 was given, since it was not specified what type of work.
Level of direct contact with sheep, cattle and goats	Never	Occasionally (at least 6 times a year), Rarely (less than 6 times a year)	Most days a week, A few days a week	Frequent contact with ruminants increases risk. So, direct contact with ruminants most days a week or a few days a week will result in a score of 2. Direct contact with ruminants occasionally or rarely will impose a much smaller risk, therefore the score is 1.
Level of direct contact with native animals	Never	Occasionally (at least 6 times a year), Rarely (less than 6 times a year)	Most days a week, A few days a week	Frequent contact with native animals increases risk. So, direct contact with native animals most days a week or a few days a week will result in a score of 2. Direct contact with native animals occasionally or rarely will impose a much smaller risk, therefore the score is 1.<br>We considered not taking this parameter into account, since other questions also addressed this and in this scoring contact with native animals is seen equally risky as contact with ruminants. However, we decided to include this question, since native animals do impose a risk.
Level of direct contact with feral animals	Never	Occasionally (at least 6 times a year),	Most days a week, A	Feral animals are likely to carry diseases and contact with this type of animals will therefore

		Rarely (less than 6 times a year)	few days a week	increase risk. Long contact results in a score of 2, short contact in a score of 1.
Level of direct contact with other animals	Never	Low risk animals	High risk animals	We manually evaluated the free form responses. We made a distinction between animals that impose a relatively low risk and animals that impose a relatively high risk. For contact with low risk animals a score of 1 was given, for contact with high risk animals a score of 2 was given. For this parameter, no participant received a risk score of 2.
Level of direct contact with pond water				We estimated that direct contact with pond water would not increase risk.
Smoking status				Smoking does not increase or decrease the risk of contracting Q-fever.
Medical history<br>Taking immune suppression medications<br>Had an MRI or CT scan in the last month<br>Received chemotherapy with a platinum-based drug<br>Other immune-modifying medical condition or treatments?				Taking immune suppression medication or undergoing other immune suppressing processes does not increase or decrease the risk of contracting Q-fever.
